# A systematic review of cognitive behavior therapy and dialectical behavior therapy for adolescent eating disorders

**DOI:** 10.1186/s40337-021-00461-1

**Published:** 2021-10-18

**Authors:** Emily N. Vogel, Simar Singh, Erin C. Accurso

**Affiliations:** 1 PGSP-Stanford PsyD Consortium, Palo Alto University, Palo Alto, CA USA; 2 Department of Psychological and Brain Sciences, Drexel University, Philadelphia, PA USA; 3 Department of Psychiatry and Behavioral Sciences, UCSF Weill Institute for Neurosciences, University of California, San Francisco, CA USA

**Keywords:** Eating disorders, Cognitive behavior therapy, Dialectical behavior therapy, Adolescent

## Abstract

**Background:**

Eating disorders have serious psychological and physical consequences. Current evidence-based treatments for adolescents with eating disorders have modest effects, underscoring the need to improve current treatment approaches. Cognitive behavior therapy (CBT) and dialectical behavior therapy (DBT) have been proposed as alternative treatment options, with burgeoning research in this area. This review aims to summarize and critically analyze the current literature on the feasibility, acceptability, effectiveness, and efficacy of CBT and DBT for adolescent eating disorders, and then proposes areas of future research.

**Methods:**

PsycINFO and PubMed were searched using the Preferred Reporting Items for Systematic Review and Meta-Analyses guidelines to identify studies examining the feasibility, acceptability, effectiveness and/or efficacy of CBT or DBT for adolescent eating disorders.

**Results:**

Eligible studies (*N* = 50; CBT: *n* = 40, DBT: *n* = 10) indicated that both treatments are reasonably feasible, acceptable, and possibly effective for adolescent eating disorders across diagnoses and levels of care, though efficacy trials are lacking.

**Conclusions:**

CBT and DBT demonstrate promise as alternatives to family-based approaches for adolescent eating disorders. Adequately powered trials to establish the effectiveness and efficacy of CBT and DBT are needed, particularly ones that compare these treatments against other leading approaches.

**Plain English summary:**

Despite high rates of relapse and likelihood for severe and enduring illness, there is a dearth of evidence-based treatment options for adolescents with eating disorders. Potentially viable but less well-studied treatments for adolescents with eating disorders include cognitive behavior therapy (CBT) and dialectical behavior therapy (DBT). This systematic review of CBT and DBT for adolescent eating disorders focuses on feasibility (i.e., how easy it was to implement the treatment), acceptability (i.e., how well the intervention was received by patients and therapists), effectiveness (i.e., how well the intervention performed under routine, real-world circumstances), and efficacy (i.e., how well the intervention performed in highly-controlled research settings). This review concludes that research supports the feasibility and acceptability of these approaches, as well as preliminary evidence of their effectiveness. However, the field is lacking studies that systematically compare CBT and DBT to other evidence-based approaches. Recommendations to advance research on CBT and DBT for adolescent eating disorders are provided, including a call for efficacy studies that clarify their performance compared to other leading approaches.

## Background

Eating disorders are associated with high rates of co-occurring psychiatric disorders [1, 2] and serious medical risks including death [3, 4]. In adolescents, some of the medical complications resulting from malnutrition (e.g., osteoporosis, growth arrest) may be irreversible if weight restoration is not attained [5]. Existing evidence-based treatments for youth with eating disorders produce modest effects at best. Indeed, over half of adolescents who receive the treatment most supported by research evidence—family-based treatment (FBT) for anorexia nervosa (AN) [6]—are still not recovered one year after completing treatment [7, 8]. Furthermore, FBT is not feasible or clinically indicated for all youth (e.g., caregiver(s) unavailable or not willing to participate, prior and/or current abuse in the home, co-occurring suicidality and emotion dysregulation that is better managed within a dialectical behavior therapy [DBT] framework). Therefore, increasing the availability of other treatments and improving treatment outcomes overall is critical.

Cognitive behavior therapy (CBT) and DBT—a third-wave CBT—have been proposed as alternative treatments that may be appropriate when FBT is not feasible. CBT is widely considered the first-line of treatment for adults with bulimia nervosa (BN) and binge eating disorder (BED) [9], and has been “enhanced” to target transdiagnostic eating disorders in adults (CBT-E: [10]) and more recently adolescents [11]. CBT may be suitable for adolescents with eating disorders given its focus on mechanisms hypothesized to maintain the disorder (e.g., overvaluation of weight and shape; [12]), efficacy for other disorders in adolescents (e.g., depression and anxiety; [13, 14]), and developmental appropriateness for adolescents (e.g., collaborative style and techniques support motivation while respecting adolescents’ developing autonomy; [15]).

Meanwhile, DBT was originally developed for adults with borderline personality disorder [16]. It incorporates both cognitive behavioral and mindfulness-based strategies in order to increase interpersonal effectiveness, improve emotion regulation, and build distress tolerance. DBT has since been applied to adolescent populations with borderline personality disorder features [17, 18] and adults with other presenting concerns [19, 20], and may be particularly appropriate for adolescents whose eating disorder psychopathology is perpetuated by mechanisms targeted in DBT, including emotion regulation (i.e., inhibited emotion expression, more typical in AN; emotion intensity and dysregulation, more typical in BN and BED; [21]). Moreover, DBT's framework is designed to focus on multiple target behaviors simultaneously, which is often crucial for adolescents with eating disorders with co-occurring disorders and related target symptoms (e.g., suicidal ideation, non-suicidal self-injury, substance abuse). The ability to focus on multiple problem areas in an integrated and structured manner—consistent with high fidelity to the treatment model—is a unique asset of DBT, in contrast to alternative, evidence-based frameworks (e.g., CBT, FBT) that require almost exclusive focus on the eating disorder, particularly early in treatment.

The present review aims to add to the literature by providing an update on second- and third-wave CBT approaches for adolescents across eating disorder diagnoses, inclusive of non-randomized study designs (i.e., case series, case studies, and naturalistic study designs). To date, there has only been one systematic review that included second- and third-wave CBTs for adolescent eating disorders, which focused exclusively on randomized controlled trials for adolescent AN or BN [22]. Data from non-randomized study designs provide important information on treatment feasibility, acceptability and effectiveness within real-world settings. In contrast, highly controlled research designs frequently fail to consider factors that impact real-world implementation (e.g., patient characteristics such as co-occurring disorders, or clinician characteristics such as level of training), creating a gap between research and clinical conditions that limit their practical value [23].

With the ultimate goal of supporting clinicians to make appropriate treatment real-world clinical decisions, studies that examine treatments in usual care settings are crucial. Therefore, this review includes studies investigating treatments with multiple components (e.g., FBT integrated with DBT skills), rather than including only studies of “pure” CBT or DBT. Although multiple third-wave CBTs have been proposed for eating disorders (e.g., acceptance and commitment therapy), we focus on DBT since it is the most widely studied to-date [24]. Given the diversity in diagnosis, treatment type, treatment setting, study methodology, and outcome measurement, a systematic review was deemed most appropriate to summarize the available data, rather than a meta-analysis, which would have yielded an effect estimate that would have been difficult to interpret given the heterogeneity of the data. Overall, this review summarizes and critically analyzes the literature on CBT and DBT for adolescent eating disorders, with the objective of clarifying current knowledge about the feasibility, acceptability, effectiveness, and efficacy of these approaches, and providing recommendations for future research.

## Methods

PsycINFO (Ovid) and PubMed were searched using the Preferred Reporting Items for Systematic Review and Meta-Analyses guidelines [25]. The following search algorithms were used, inclusive of all papers published through December 2020: [(cognitive behavior therapy OR CBT) AND (adolesc*) AND (eating disorder)], and [(dialectical behavior therapy OR DBT) AND (adolesc*) AND (eating disorder)]. The first author screened CBT titles and abstracts for relevance, and the second author screened DBT titles and articles and abstracts for relevance. Articles deemed relevant based on title and abstract were then reviewed. Each author replicated and verified the other author’s search, to control for bias. Discrepancies were discussed and resolved in consultation with the senior author. Eligible articles (1) assessed the feasibility, acceptability, effectiveness and/or efficacy of CBT, DBT, or any other psychological treatment based at least in part on CBT or DBT (2) in adolescents with eating disorders (3) and were published in peer-reviewed, academic journals (4) in the English language. Studies that assessed interventions in a partial hospitalization setting (*n* = 2 CBT studies: one for AN and one for avoidant/restrictive food intake disorder, *n* = 3 DBT studies: one for restrictive eating disorders and two for transdiagnostic eating disorders) were excluded because there were too few studies by treatment type and diagnostic category within this setting type to draw meaningful conclusions.

Variables extracted from studies included sample characteristics (sample size, mean age, diagnoses); treatment attrition rate; treatment characteristics (treatment type, setting, length); study follow-up time-points; variables related to eating disorder psychopathology, behavior, and weight restoration; additional primary outcomes relevant to the effectiveness or efficacy of the intervention; and any outcome variables related to the feasibility or acceptability of the intervention. The study results are grouped by intervention type (CBT, DBT) and level of care (e.g., outpatient, inpatient) (see Tables 1, 2), then organized by eating disorder diagnosis in the results section below. Given the inclusion of non-randomized designs, our conclusions are based on a qualitative synthesis of the data.

**Table 1:** Cognitive behavior therapy trials for adolescent eating disorders

Authors (year)	Study design	Population		Therapy (*n*)	Attrition (%)	Tx length	FU	Summary of relevant findings	Quality appraisal
		Dx(s)	Age *M* [range]						
*CBT (outpatient settings)*									
Ball and Mitchell (2004) [37]	RCT	AN	18.5 [13–23]	CBT (13) versus behavioral family therapy (12)	CBT: 30.8 Behavioral family therapy: 25	21–25 sessions over 12 mo	6 mo	No differences between treatment groups at EOT or FU in weight, return of menses, or ED psychopathology. At EOT, 77.8% (*n* = 7) of each treatment group achieved min. weight gain of 4 kg (8.8 lbs) and within 10% of adolescent’s average body weight, with binge and purge episodes occurring less than once per week. Improvements were maintained at FU. Statistically significant improvement in ED psychopathology and behavior (EDE, EDI-2, ABOS) for both treatment groups at EOT, but all measures remained in the symptomatic range at all FU timepoints. Of patients that completed either treatment (*n* = 18), 72% achieved min. weight gain of 4 kg (8.8 lbs) and within 10% of adolescent’s average body weight, return of menses, and abstinence from binge and purge episodes at 6mo FU	Fair^a^
Byford et al. (2007)^†^ [34]	Secondary analysis of RCT data (Gowers et al., 2007; [82])	AN	14.9 [12–18]	CBT w/parent counseling, dietary therapy (55) versus multi-disciplinary inpatient treatment (57) versus general community care (55)	CBT: 25.5 Inpatient: 50.9 General community care: 30.9	CBT: 6 mo Inpatient: 6 wks General community care: 6 mo	1 yr 2 yr	The outpatient CBT group spent the least amount of time in the hospital compared to the other two treatment groups. There were no statistically significant differences in cost of treatment between the three treatment groups, though the CBT group was least expensive at non-significant level	Fair^a^
Charpentier et al. (2003) [41]	Cohort study	AN BN	17.7 [13–22]	Group CBT (26)	12	13 wks (≈ 3 mo)	3 mo	For patients with BN, statistically significant reduction in ED psychopathology (EDI) and frequency of binges and purging at EOT, which was maintained at 3mo FU. For patients with AN, statistically significant increase in BMI, with no significant changes in other ED symptoms (EDI)	Fair^a^
Cibich and Wade (2019) [77]	Case study	BN	16 [N/A]	CBT-T (1)	N/A	10 sessions	3 mo	Abstinence from binge eating and purging by second session, and ED psychopathology (EDE-Q) within community norm range by EOT. All changes maintained at 3mo FU	Include^b^
Cowdrey and Davis (2016) [78]	Case study	AN	15 [N/A]	CBT-E (1)	N/A	11 wks (≈ 3 mo)	8 mo	Marked reduction in ED behaviors and "feeling fat" (self-monitoring records), global ED psychopathology (EDE-Q) within community norms, and clinically significant increases in weight at EOT. At 8 mo, progress was partially maintained (global EDE-Q score had increased to just above clinical cut-off)	Include^b^
Craig et al. (2019) [79]	Cohort study	AN, atypical AN, BN, atypical BN	15.5 [13–18]	CBT-ED (54)	38.9	Varied (*M* = 22 wks; range: [6–46 wks], or *M* ≈ 5 mo, range: [1–10 mo])	None	Statistically significant improvement in ED psychopathology (EDE-Q) at EOT (*d* = 0.82). For patients with AN or atypical AN, statistically significant increases in mean %EBW (AN: *d* = 0.61; atypical AN: *d* = 0.36)	Fair^a^
Dalle Grave et al. (2013) [35]	Cohort study	AN	15.5 [13–17]	CBT-E (46)	17.4	40 wks (≈ 9 mo)	60 wk (≈ 14 mo)	Nearly two thirds (63%, *n* = 29) completed 40 sessions without need for additional treatment. Of treatment completers, 31% (*n* = 9) reached 95% EBW by EOT. Nearly all treatment responders (97%, *n* = 28) had global ED psychopathology (EDE-Q) within community norms at EOT. Changes remained stable at 60wk FU	Good^a^
Dalle Grave et al. (2015) [48]	Cohort study	BN, BED, or other specified ED	16.5 [13–19]	CBT-E (68)	25	20 wks (≈ 5 mo)	None	Statistically significant improvements in ED psychopathology (EDE-Q) at EOT (*d* = 1.03). More than half (67.6%, *n* = 46) had minimal residual ED psychopathology (global EDE-Q scores below 1 SD above the community mean) at EOT. Of participants with binge eating or purging at BL, 50% (*n* = 25) were abstinent at EOT. Of treatment completers, 81.2% (*n* = 42) achieved minimal residual ED psychopathology at EOT, and 76.5% (*n* = 26) of those with binge eating or purging at BL were abstinent at EOT	Good^a^
Dalle Grave et al. (2019) [31]	Cohort study	AN	15.5 [11–18]	CBT-E (49)	28.6	40 wks (≈ 9 mo)	20 wk (≈ 4 mo)	The majority of treatment completers (62.9%, *n* = 22) achieved both good weight outcome (corresponding to an adult BMI ≥ 18.5 kg/m^2^) and global ED psychopathology (EDE-Q) within community norms at EOT. Nearly half of patients that completed the 20wk FU (48.3%, *n* = 14) maintained this outcome	Good^a^
DeBar et al. (2013) [30]	RCT	BED, BN	15.12 [12–18]	CBT (13) versus TAU (12)	23 (CBT) 30 (TAU)	8 wks + 4 optional wks (≈ 2–3 mo)	3 mo 6 mo	At FU, CBT held advantage over TAU, with statistically significant higher rates of abstinence from binge eating (at 6mo FU, *d* = 1.47), and more improvements in eating, shape and weight concerns (at 6mo FU, EDE Eating Concerns *d* = 0.80, EDE Shape Concerns *d* = 1.04, EDE Weight Concerns *d* = 0.64). 100% of CBT participants were abstinent from binge eating at 6mo FU. Participants in the CBT group reported high post-treatment satisfaction (Client Satisfaction Survey)	Fair^a^
Gorrell et al. (2019)^†^ [80]	Secondary analysis of RCT data (Le Grange et al., 2015; [44])	BN, BN-type EDNOS (DSM-IV)	15.8 [12–18]	FBT-BN (52) versus CBT-A (58)	10	Varied (FBT-BN, *M* = 13.6 wks; CBT-A, *M* = 14.7 wks) (both ≈ 3 mo)	none	Across both treatments, participants with a higher level of motivation to change in ED-related preoccupations and rituals (Motivation for Change subscale of YBC-EDS) at BL were more likely to have reduced ED psychopathology (EDE global score) at EOT. Motivation to change ED-related preoccupations and rituals at BL had no effect on abstinence from bingeing and purging at EOT in either treatment	Good^a^
Gowers and Smyth (2004) [81]	Cohort study	AN	16.1 [12–20]	CBT + parental counseling + dietary therapy (42)	21.5	12 sessions CBT, 4 sessions parental counseling, 3 sessions dietary counseling	6 wk	At 6wk FU, significant improvements in ED psychopathology (EDI-2) and weight gain. Those with higher motivation (assessed using survey designed by research team) were more likely to complete treatment. Motivational status did not predict self-rated outcome (including EDI-2) at FU, though higher level of motivation at BL predicted more weight gain at 6wk FU	Fair^a^
Gowers et al. (2007) [82]	RCT	AN	14.9 [12–18]	CBT w/parent counseling, dietary therapy (55) versus multi-disciplinary inpatient treatment (57) versus general community care (55)	CBT: 25.5 Inpatient: 50.9 General community care: 30.9	CBT: 6 mo Inpatient: 6 wks General community care: 6 mo	1 yr 2 yr	At 1 yr FU, mean improvement in weight and ED psychopathology (EDI-2, MRAOS) across treatment groups, with no statistically significant differences between them. At 2 yr FU, further improvement in outcome for all groups, with no statistically significant differences between them	Fair^a^
Gowers et al. (2010) ^†^ [32]	Secondary analysis of RCT data (Gowers et al., 2007; [82])	AN	14.9 [12–18]	CBT w/parent counseling, dietary therapy (55) versus multi-disciplinary inpatient treatment (57) versus general community care (55)	CBT: 25.5 Inpatient: 50.9 General community care: 30.9	CBT: 6 mo Inpatient: 6 wks General community care: 6 mo	1 yr 2 yr 5 yr	At 5 yr FU, trend of improvement across treatment groups in ED outcome (MRAOS), and no statistically significant differences between groups in % weight for height, diagnostic outcome, or ED psychopathology (EDI-2, MRAOS). No significant differences in total cost of hospital use across treatment groups at 5 yr FU. No statistically significant differences between groups in parental expectations of treatment (at 1 yr FU, self-reported rating of prior expectation of treatment received on 7-point Likert scale), but adolescents’ expectations of general outpatient care were lower than of CBT outpatient care. At 1 yr FU, parents reported satisfaction across treatments (rated on 7-point Likert scale), but were significantly more satisfied with CBT outpatient care compared to general community care. Adolescents had significantly lower satisfaction with care across treatments compared to parents, though a non-significant trend favored adolescent satisfaction with CBT outpatient care compared to general community care	Fair^a^
Hilbert et al. (2020) [47]	RCT	BED	15.3 [12–20]	CBT (32) vs WLC (36)	32	20 sessions over 4 mo	6 mo 12 mo 24 mo	Compared to WLC group, CBT group had significantly higher rates of abstinence from binge eating (51% vs. 33%) and remission from BED (57% vs. 33%) and significantly less ED psychopathology at EOT (EDE). CBT group maintained its advantage relative to WLC in in ED behaviors and psychopathology at all FU timepoints	Good^a^
Hurst et al. (2017) [83]	Case study	BN	15 [15–15]	FBT-BN (1) versus FBT-BN + CBT (1)	0	Not reported	None	Participants in both treatments achieved full remission at EOT (EDE global score within 1 SD of community norms). Participant 1 reduced binge and purge episodes from an average of 12 per week at BL, to 100% abstinence at EOT. Participant 2 reduced binge and purge episodes from an average of ten per week at BL, to 100% abstinence at EOT	Include^b^
Hurst and Zimmer-Gembeck (2019) [36]	Cohort study	AN	14.9 [12–17]	FBT + CBT-P (21)	9	Varied (*M* = 32 wks; 23 FBT sessions and 9 CBT sessions) (≈ 7 mo)	None	Over half (57%; *n* = 12) of adolescents achieved full remission by EOT, defined as a minimum of 95% of EBW and global EDE-Q score within community norms. Two participants demonstrated increased ED symptoms at EOT	Good^a^
Jaite et al. (2018) [33]	RCT	AN	16.9 [12–21]	CBT (24) vs DBT (26)	Not reported	25 wks (wkly individual and group sessions) (≈6 mo)	None	ED symptoms (EDI-2) decreased and BMI increased significantly from BL to EOT in both treatments (CBT: EDI-2 *d* = -0.61, BMI *d* = 1.04; DBT: EDI-2 *d* = -0.55, BMI *d* = 0.7). Treatment assignment (CBT vs DBT) did not predict treatment satisfaction. Significant correlation between therapist and parent level of satisfaction of treatment (Questionnaire for the Evaluation of Treatment). Agreement was not observed between adolescent and therapist, nor adolescent and parent level of satisfaction in treatment	Fair^a^
Le Grange et al. (2015) [44]	RCT	BN, BN-type EDNOS (DSM-IV)	15.8 [12–18]	FBT-BN (52) versus CBT-A (58)	10	Varied (FBT-BN *M* = 13.6 wks; CBT-A *M* = 14.7 wks) (both ≈ 3 mo)	6 mo 12 mo	Abstinence from binge eating and purging was significantly higher in FBT-BN than CBT-A at EOT and 6mo FU. Abstinence from these behaviors was no longer significantly different between the groups at 12mo FU. No significant differences between groups in %EBW, EDE global score, YBC-EDS total score	Good^a^
Le Grange et al. (2020) [49]	Cohort study	Any DSM-5 ED diagnosis, excluding ARFID	14.6 [12–19]	FBT (51) versus CBT-E (46)	FBT: 35 CBT: 37	FBT: 20 sessions over 6 mo CBT: 40 sessions over 9–12 mo for lower weight pts; 20 sessions over 6 mo for higher weight pts	6 mo 12 mo	Rate of weight gain was faster in FBT than CBT by EOT, though differences in weight gain were no longer significant at 6 and 12mo FUs. There were no differences in ED psychopathology (EDE global score) between the treatments at any time point	Good^a^
Lock (2005) [43]	Cohort study	BN	15.8 [12–18]	CBT (34)	18	Varied (*M* = 15.8 sessions) (≈ 4mo)	None	Majority of patients (82%, *n* = 28) completed at least 10 weeks of treatment. The mean rate of binge eating and purging reduced from 15.8 episodes/wk (range: [2–21]) at BL to 3.4 episodes/wk (range: [0–21]) at EOT. Over half of participants (56%, *n* = 19) were abstinent from binge eating and purging at EOT	Fair^a^
Matheson et al. (2020)^†^ [84]	Secondary analysis of RCT data (Le Grange et al., 2015; [44])	BN, BN-type EDNOS (DSM-IV)	15.7 [12–18]	CBT-A (26) vs FBT-BN (30) vs SPT (15)	See Le Grange et al. (2015)	Varied (*M* = 14 sessions) (≈ 3 mo)	6 mo 12 mo	Reducing purge episodes by ≥ 96.8% by session 2 and reducing binge eating episodes by ≥ 96.4% by session 4 predicted abstinence in these behaviors at EOT, regardless of treatment type. Reducing binge eating episodes by ≥ 96.4% by session 8, and purge episodes by ≥ 94.4% by session 9 predicted abstinence at 6mo FU, and reductions in binge eating by ≥ 96.4% at session 9 predicted abstinence at 12mo FU, regardless of treatment type	Good^a^
Ohmann et al. (2013) [85]	Cohort study	AN	14.3 [13–17]	Group CBT (29)	28	40 wkly group sessions + monthly family sessions (≈ 9 mo)	1 yr	Over half of patients (55%, *n* = 16) achieved good outcome, defined as reaching the 25^th^ age-related BMI percentile, and absence of restriction or bulimic behaviors. At EOT, weight and BMI improved significantly in patients with good outcome and remained stable at 1 yr FU. There was little to no improvement in weight in patients with poor outcome or who dropped out of treatment (44.8%, *n* = 13). Patients who achieved good outcome also demonstrated statistically significant improvement in ED psychopathology, per EDE dietary restraint, eating concern and weight concern subscales, though improvements were not observed in the EDE shape concern subscale	Fair^a^
Puls et al. (2019)^†^ [46]	Secondary analysis of RCT data (Hilbert et al., 2020; [47])	BED	14.17 [12–20]	CBT (64)	32	20 wkly sessions (≈ 5 mo)	None	High levels of therapist treatment adherence (ACF) and therapeutic alliance (WAI-OS) observed across all sessions. Decreased adherence was associated with higher patient treatment expectation (VAS rating of expectation of treatment success). No association was observed between treatment adherence and ED outcomes (# OBE and SBE episodes, EDE global score). Alliance was negatively associated with # OBE and SBE episodes, and positively associated with treatment adherence. There was no association between alliance and general ED psychopathology (EDE) or patient treatment expectation	Good^a^
Schapman-Williams et al. (2006) [42]	Cohort study	BN, BN-type EDNOS	16.3 [range not reported]	CBT (7)	0	Varied (*M* = 15.3 sessions over 5.4 mo; range: [10–20 sessions over 4–8 mo])	None	At EOT, significant reduction in ED psychopathology (EDE), and mean # binge episodes/wk. Reduction in mean # purge episodes/wk approached statistical significance at EOT, and 57% (*n* = 4) were abstinent from both binge eating and purging	Fair^a^
Schapman-Williams and Lock (2007) [86]	Case study	BN	16	CBT (1)	N/A	20 sessions (≈ 5 mo)	None	Consistent normalization of eating patterns by session six (assessed via daily food records). Abstinence from binge and purge episodes maintained throughout treatment, aside from one episode in week 13 (a reduction from > 20 binge and purge episodes/wk pre-treatment). Distress while eating reduced from 60% at session one to 0% by session nine. ED psychopathology (EDE) reduced substantially by mid-treatment, which was maintained at EOT	Include^b^
Stefini et al. (2017) [39]	RCT	BN, BN-type EDNOS (DSM-IV)	18.7 [14–20]	CBT (39) versus PDT (42)	38.5 (CBT) 21.4 (PDT)	Varied (*M* = 36.6 sessions or ≈ 8 mo; up to 60 sessions over 1 yr)	12 mo	No significant differences in attrition, diagnostic changes or reduction in ED psychopathology at EOT or 12mo FU between groups. Significant reductions in binge and purge frequency, and ED symptoms (EDE, EDE-Q) in both groups at EOT, which were maintained at 12mo FU. CBT showed a small advantage over PDT in reduced frequency of binge eating (*d* = 0.23) and purging (*d* = 0.26) at EOT (EDE-Q), while PDT showed a small advantage over CBT in eating concern (*d* = -0.35) at EOT (EDE)	Fair^a^
Sysko and Hildebrandt (2011) [87]	Case study	BN-type EDNOS	16 [N/A]	CBT-E (1)	N/A	29 sessions over 9 mo	None	Clinically significant decrease in SBEs and purging in first 4 weeks. Abstinence from SBEs and purging achieved by session 22. Residual concerns around shape and weight still present at EOT	Include^b^
Thompson-Brenner et al. (2010) [50]	Observational cross-sectional study	AN, BN, EDNOS	16.4 [15–18]	CBT, dynamic therapy, family intervention, emotion regulation, trauma therapy or conjoint therapy (120; *n* per treatment type not reported	N/A	Varied (*M* = 8 mo, range: [6 h–1 yr])	None	Dynamic therapy was most strongly associated with better global outcome for the entire sample, though CBT was most strongly associated with better outcome for a subsample of participants with poor relational/personality functioning (assessed via clinician rated psychotherapy effectiveness form)	Good^a^
Valenzuela et al. (2018) ^†^ [45]	Secondary analysis of RCT data (Le Grange et al., 2015; [44])	BN, BN-type EDNOS (DSM-IV)	15.8 [12–18]	FBT-BN (52) versus CBT-A (58)	10	Varied (FBT-BN *M* = 13.6 wks; CBT-A *M* = 14.7 wks) or (both ≈ 3 mo)	6 mo 12 mo	Statistically significant and clinically meaningful reduction in symptoms of depression (BDI) and self-esteem (RSE) at FU in both treatments, with no statistically significant differences between them	Good^a^
*CBT guided self-help*									
Perkins et al.(2005)^†^ [54]	Secondary analysis of RCT data (Schmidt et al., 2007; [51])	BN, BN-type EDNOS	17.9 [13–20]	CBT guided self-care or Family therapy adapted from FBT (85)	Not reported	Not reported	None	Both treatment groups included opportunities for parental involvement; for present analyses, total sample grouped into “No Parent Involvement” (*n* = 23) vs “Parent Involvement” (*n* = 62). Most common reasons participants reported choosing to exclude parents from treatment were lack of comfort discussing personal issues in their presence, feeling that the ED was “their problem” and parents didn’t need to be involved, or another personal factor unique to the adolescent (e.g., parents not thinking the adolescent had a problem). The most common reasons adolescents reported wanting parents to be involved in treatment included that the parent was viewed as supportive, had time for the patient, was interested in the patient and wanted to be involved, and the patient wanted them to learn more about the ED	Fair^a^
Pretorius et al. (2009) [52]	Cohort study	BN, BN-type EDNOS	18.8 [13–20]	Online CBT guided self-care (101)	17	8 web-based sessions, encouraged to complete wkly	3 mo 6 mo	Significant improvements in bulimic behaviors and cognitions at 3mo and 6mo FUs (EDE, EDE-Q) although most remained symptomatic; of treatment completers, 10% (*n* = 5) were abstinent from bulimic behaviors at 3mo FU, and 17% (*n* = 11) were abstinent from bulimic behaviors at 6mo FU. High patient satisfaction with treatment convenience, acceptability, and confidentiality (assessed via questionnaire designed by research team)	Good^a^
Pretorius et al. (2010) ^†^ [53]	Secondary analysis of cohort study data (Pretorius et al., 2009; [52])	BN, BN-type EDNOS	*M* not reported; 16–19: *n* = 44; 20 years: N *n* = 7 [16–20]	Online CBT guided self-care (11)	N/A	8 web-based sessions. At time of interview, # sessions completed ranged from one to eight	None	Results included qualitative data regarding liked and disliked components of the web-based, guided self-care program. Participants found the layout and presentation of the program components to be user-friendly, and reported their choice of using a web-based program was influenced by accessibility, flexibility, sense of control over treatment, anonymity, non-judgmental nature, privacy, and convenience. Most participants felt that the program offered sufficient support, felt motivation to change at the start of treatment, motivation difficulties toward the end, and described improvement in ED symptoms that they attributed to the program	Good^a^
Schmidt et al. (2007) [51]	RCT	BN, BN-type EDNOS	17.7 [13–20]	CBT guided self-care (44) vs Family therapy adapted from FBT (41)	30	CBT: 22 sessions, Family therapy: 24 sessions (both ≈ 5 mo)	6 mo 12 mo	Both interventions demonstrated significant improvements in binge eating and purging behaviors (EATATE interview) across time. A significantly higher proportion of participants were abstinent from binge eating at 6mo FU in the CBT guided self-care group compared to the family therapy group. These differences were no longer significant at 12mo FU. There were no significant differences in change in purging behaviors between the two groups at 6mo or 12mo FU. Mean cost of treatment significantly lower for CBT guided self-care than family therapy (Client Service Receipt Inventory)	Fair^a^
Wagner et al. (2013) [55]	RCT	BN	19.3 [16–21]	Online CBT-based guided self-help (18) versus guided self-help biblio-therapy (11)	31	Varied (*M* not reported; range: [4–7 mo])	4 mo 7 mo 18 mo	No differences in outcome between treatment groups; outcomes reported combines data from the two groups. Significant improvement over time in monthly binge eating, vomiting and fasting. At 7mo and 18mo FU, about half of adolescents (44%, *n* = 8 and 55%, *n* = 11, respectively) were abstinent from ED behaviors or achieved remission, with outcomes comparable to an adult sample receiving the same treatments. There were no significant improvements in the EDI-2 perfectionism scale, and asceticism EDI-2 subscale scores increased slightly between 4- and 7mo FU. All other EDI-2 subscales demonstrated improvement over time, with the highest decrease in scores within the first 4 mo of therapy	Poor^a^
*CBT-based inpatient care*									
Calugi and Dalle Grave (2019) [56]	Cohort study	AN	16.4 [13–18]	CBT-E (62)	9.7	20 wks (≈ 5 mo)	6 mo 12 mo	Significant mean reduction in body image concerns (EDE), and nearly all treatment completers (96.4%) reached a normative BMI percentile at EOT (corresponding to adult BMI ≥ 18.5 kg/m^2^). At 6mo and 12mo FU, 78.7% and 80.4% of treatment completers maintained a normative BMI, respectively	Good^a^
Dalle Grave et al. (2014) [57]	Cohort study	AN	16.0 [13–17]	CBT-E (27)	3.7	20 wks (≈ 5 mo)	6 mo 12 mo	Treatment was well-accepted by patients, demonstrated by low attrition. Nearly all treatment completers (96.2%, n = 25) reached a normative BMI (corresponding to a BMI percentile ≥ 18.5) by EOT. Statistically significant decrease in ED psychopathology (EDE) by EOT, and 38.5% (*n* = 10) had minimal residual ED psychopathology (EDE global score below 1 SD above community norms). Changes were maintained at 6mo and 12mo FU	Good^a^
Fennig et al. (2017) [58]	Cohort study	AN	14.8 [11–18]	Multimodal IP program, with CBT component (44)	29.5	Varied (*M* ≈ 4 mo, range [12 days–≈ 9 mo])	None	BMI significantly increased from admission to discharge, with 70% achieving 100% IBW at discharge, and 25% achieving 90% IBW. Significant improvement in general ED psychopathology severity (EDI-2 total score) and restraint and eating concern subscales, but no significant change in body dissatisfaction, weight concern, drive for thinness, or shape concern subscales	Fair^a^
Naab et al. (2013) [59]	Cohort study	AN	16.4 [13–17]	Multimodal IP program, with CBT component (177)	30.5	Varied (*M* = 77.9 days) (≈ 3 mo	None	Significant mean increases in BMI and decrease in global ED psychopathology (SIAB-S global score) from admission to discharge. Compared to a sample of adults receiving the same treatment, differences in outcome were only found in the bulimic symptoms and atypical binge subscales of the SIAB-S, with adults showing greater severity at admission, and greater improvement upon discharge	Fair^a^
Schlegl et al. (2016) [60]	Cohort study	AN	15.7 [13–17]	Multimodal IP program, with CBT component (238)	19.8	Varied (*M* = 81.9 days) (≈ 3 mo)	None	Significant increases in BMI from admission to discharge (*d* = 2.1), and improvement in ED psychopathology (EDI-2) at discharge (*d* = 0.8), with 44.7% demonstrating a clinically significant improvement, though 3.7% deteriorated	Good^a^

The quality appraisal tools are denoted with a superscript (^a^NIH Quality Assessment Tools and ^b^Joanna Briggs Institute Critical Appraisal Checklist for Case Reports)

ABOS = Anorectic Behavior Observation Scale; ACF = Adherence Control Form; AN = anorexia nervosa; ARFID = avoidant/restrictive food intake disorder; BDI = Beck Depression Inventory; BED = binge eating disorder; BL = baseline; BMI = body mass index; BN = bulimia nervosa; CBT = cognitive behavioral therapy; CBT-A = CBT adapted for adolescents; CBT-ED = CBT for eating disorders; CBT-E = Enhanced CBT; CBT-P = CBT Perfectionism modules; CBT-T = ten session CBT; DBT = dialectical behavior therapy; DSM = Diagnostic and Statistic Manual of Mental Disorders; Dx = Diagnosis; EBW = expected body weight; ED = eating disorder; EDE = Eating Disorder Examination; EDE-Q = Eating Disorder Examination-Questionnaire; EDI = Eating Disorder Inventory; EDNOS = eating disorder not otherwise specified; EOT = end of treatment; FBT = family-based treatment; FU = follow-up; IBW = ideal body weight; IP = inpatient; mo = month; MRAOS = Morgan Russell Average Outcome Scale; N/A = not applicable; OBE = objective binge episode; PDT = psychodynamic therapy; RCT = randomized controlled trial; RSE = Rosenberg Self-Esteem Scale; SBE = subjective binge episode; SIAB-S = Structured Interview for Anorexic and Bulimic Syndromes; SPT = supportive psychotherapy; TAU = treatment as usual; Tx = treatment; VAS = visual analogue scale; WAI-OS = Working Alliance Inventory-shortened, observer-rated version; wk = week; WLC = wait list control; YBC-EDS = Yale-Brown-Cornell Eating Disorder Scale; yr = year

^†^Secondary analysis paper, using data from an original trial represented in the review

**Table 2:** Dialectical behavior therapy trials for adolescent eating disorders

Authors (year)	Study design	Population		Therapy (*n*)	Attrition (%)	Tx length	FU	Summary of relevant findings	Quality appraisal
		Dx(s)	Age *M* [range]						
*DBT (outpatient settings)*									
Accurso et al. (2018) [62]	Cohort study	AN	15.5 [11–18]	FBT + DBT skills (11)	27.3	19 sessions over 19 wks (≈ 4 mo)	None	By EOT, 36.4% (*n* = 4) of participants were weight-restored, with significant increase in weight over treatment (*d* = 1.39); 54.5% (*n* = 6) reported an EDE global score within 1 SD of community norms; and 18.2% (*n* = 2) achieved both weight restoration and EDE Global scores within community norms. Decrease in ED pathology was corroborated by parent accounts (PEDE; *d* = 0.55). Small improvements in distress tolerance (DTS; *d* = 0.14) and emotion regulation (DERS; *d* = 0.14) were also demonstrated. Of those with secondary amenorrhea (*n* = 5), 100% resumed menses during the course of treatment. Treatment was perceived as “appropriate” and “acceptable” by all patients and their families (TSPE). Patients reported therapeutic alliance as moderate, while parents rated it highly (HAq)	Fair^a^
Burton et al. (2020) [64]	Case study	BED	18 [N/A]	DBT skills group (1)	0	10 sessions over 10 wks (≈ 2 mo)	None	Throughout treatment, statistically significant decline in urges to engage in binge eating and mindless eating, with abstinence from mindless eating by week 2. At post-treatment, clinically significant improvement in depressive symptoms (*RCI* = 3.13), but no change in anxious or somatic symptoms. Decrease in binge eating was observed by EOT (BES), but not statistically significant (*RCI* = 1.64). Patient reported that radical acceptance was a particularly useful DBT skill	Include^b^
Fischer and Peterson (2015) [61]	Cohort study	BED, EDNOS	16.2 [14–17]	Full-model DBT (10)	30	6 mo	6 mo	At post-treatment, seven treatment completers reported significantly reduced self-harm (DSHQ; *d* = 1.35); frequency of OBEs (*d* = 0.46), purging episodes (*d* = 0.66); and global eating psychopathology (EDE; *d* = 0.64). At FU, 85.7% of participants (*n* = 6/7) were abstinent from self-injury (DSHQ); 50.0% of participants (*n* = 3/6) were abstinent from binge eating; and 66.7% of participants (*n* = 2/3) were abstinent from purging. EDE scores continued to improve through follow-up (*d* = 1.13)	Fair^a^
Kamody et al. (2019) [65]	Cohort study	BED	15.4 [14–18]	DBT skills group (15)	0	10 sessions over 10 wks (≈ 2 mo)	None	Patients meeting criteria for BED decreased from BL (*n* = 6, 40%) to EOT (*n* = 3, 20%); and among those who completed follow-up, only 1 individual (9.1%) met criteria for BED. Youth and caregiver reports showed reductions in emotional eating (EES-C) and binge-eating post-treatment (EDE-Q). Acceptability ratings were high among treatment completers: 86.7% (*n* = 13) reported they would be willing to participate again; 93.3% (*n* = 14) reported they would suggest the intervention to someone else with eating problems; all participants (100%, *n* = 15) reported feeling confident in their ability to use learned skills to combat emotional overeating	Fair^a^
Kamody et al. (2020) ^†^ [67]	Secondary analysis of cohort study data (Kamody et al., 2019; [65])	BED	15.4 [14–18]	DBT skills group (15)	0	10 sessions over 10 wks (≈ 2 mo)	None	Statistically significant increases in distress tolerance appraisal (DTS; *d* = 0.23), cognitive reappraisal (ERQ-CA; *d* = 0.38), and expressive suppression (ERQ-CA; *d* = 0.27) from BL to EOT. DBT was evaluated as both acceptable and feasible by patients (DBT-SRS), with radical acceptance possessing the highest mean rating, followed by mindful eating and three mind states	Fair^a^
Mazzeo et al. (2016) [70]	RCT	BED, LOC eating	15.4 [13–17]	DBT skills group (28), versus weight mgmt. group (17)	31.1	Wave 1 = 12 wkly sessions (≈ 3 mo); waves 2–5 = 8 wkly sessions (≈ 2 mo)	None	Feasibility rated highly by therapists (therapist feasibility questionnaire), and feasibility and acceptability rated highly by patients (participant satisfaction questionnaire) for both conditions; neither condition was superior to the other. Statistically significant improvements in EDE-Q global scores, and eating, shape, and restraint subscales by EOT for both conditions; again, neither condition was superior to the other. Negative affect (EES-C) and EDE-Q weight concerns significantly improved by EOT, only for the weight management group. No changes in eating in the absence of hunger (EAH-C) for either condition	Poor^a^
Murray et al. (2015) [63]	Cohort study	BN	15.7 [14–17]	FBT + DBT skills (35)	0	Varied (*M* = 77.2 days) (≈ 3 mo)	None	From intake to discharge, significant improvements in EDE-Q global eating scores, OBEs, SBEs, vomiting episodes, and DERS emotion regulation strategies. Improvements in parental efficacy were observed at EOT (PVA). No improvements in the remaining DERS subscales, EDE restraint and eating concerns, or BMI	Good^a^
Peterson et al. (2020) [68]	Cohort study	AN, OSFED	15.3 [13–18]	FBT + DBT skills (18)	33.3	26 sessions over 6 mo	None	From BL to EOT, large effect sizes for increases in adaptive skills (DBT-WCCL; *d* = 0.71) and decreases in dysfunctional coping strategies (DBT-WCCL; *d* = 0.85); medium effect sizes for decreases in binge eating (*d* = 0.40) and increase in %EBW (*d* = 0.32); and small effect sizes for decreases in EDE-Q global scores (*d* = 0.26), EDE-Q restraint subscales (*d* = 0.29), and CDI scores (*d* = 0.28). Post-treatment, 92% of the participants were abstinent from OBEs, compared to 75% pre-treatment	Fair^a^
Safer et al. (2007) [66]	Case study	BED	16 [N/A]	DBT-informed individual therapy (1)	0	21 sessions over 21 wks (≈ 5 mo)	3 mo	Reduction in OBEs over the past 28 days, from 22 episodes pre-treatment, to 4 episodes post-treatment. Substantial reduction in EDE restraint subscale, though EDE weight, shape, and eating concerns either increased or remained the same. 3 lb weight gain by EOT. Only 1 OBE was reported 1 mo post-treatment, with no OBEs over the last 2 mos of FU	Include^b^
Salbach-Andrae et al. (2008) [69]	Cohort study	AN, BN	16.5 [12–18]	Full-model DBT (12)	8.3	50 sessions over 25 wks (≈ 6 mo)	None	Significant improvements in ED symptoms and global ED psychopathology post-treatment, with reductions on all EDI-2 subscales (*d* range: 0.43–1.10). Significant reduction in the Global Severity Index of the SCL-90-R by EOT (*d* = 0.57). Of the 11 treatment completers, 45.5% (*n* = 5) no longer met criteria for an ED by EOT. Over half (54.5%, *n* = 6) of treatment completers reported reduction in vomiting frequency (*d* = 1.7); all patients (100%; *n* = 5) who reported binge eating at BL were abstinent at EOT (*d* = 1.9); and 81.8% (*n* = 9) of treatment completers reported reduction in food restriction (*d* = 1.2). Significant increases in BMI for patients with AN (*d* = 2.60)	Fair^a^

The quality appraisal tools are denoted with a superscript (^a^NIH Quality Assessment Tools and ^b^Joanna Briggs Institute Critical Appraisal Checklist for Case Reports)

AN = anorexia nervosa; BED = binge eating disorder; BES = Binge Eating Scale; BL = baseline; BMI = body mass index; BN = bulimia nervosa; CDI = Child Depression Inventory; DBT = dialectical behavior therapy; DBT-SRS = DBT Skill Rating Scale; DBT-WCCL = DBT Ways of Coping Checklist; DERS = Difficulties in Emotion Regulation Scale; DSHQ = Deliberate Self Harm Questionnaire; DTS = Distress Tolerance Scale; Dx = diagnosis; EAH-C = Eating in the Absence of Hunger Questionnaire for Children and Adolescents; EBW = expected body weight; ED = eating disorder; EDE = Eating Disorder Examination; EDE-Q = Eating Disorder Examination-Questionnaire; EDI-2 = Eating Disorder Inventory-2; EDNOS = eating disorder not otherwise specified; EES-C = Emotional Eating Scale for Children and Adolescents; EOT = end of treatment; ERQ-CA = Emotion Regulation Questionnaire for Children and Adolescents; FBT = family-based treatment; FU = follow-up; HAq = Helping Alliance questionnaire; LOC = loss of control; mo = month; OBE = objective binge episode; OSFED = other specified feeding or eating disorder; PEDE = Parent Eating Disorder Examination; PVA = Parents Versus Anorexia Scale; RCT = randomized controlled trial; SBE = subjective binge episode; SCL-90-R = Symptom Checklist-90 Revised; TSPE = Therapy Suitability and Patient Expectations; Tx = treatment; wk = week

Unspecified EDs classified as either EDNOS or OSFED, depending on which version of the DSM was available when the study was published

^†^Secondary analysis paper, using data from an original trial represented in the review

Risk of bias for each study was evaluated using study quality assessment tools recommended in a recent review [26], published by the National Institute of Health [27] and the Joanna Briggs Institute [28]. Cohort studies (i.e., longitudinal studies sampled based on exposure to the intervention rather than outcome; [29]) with no control group were rated using the *NIH Quality Assessment Tool for Before-After (Pre-Post) Studies with No Control Group*. Randomized controlled trials were rated using the *NIH Quality Assessment of Controlled Intervention Studies*. The *NIH Quality Assessment Tool for Observational Cohort and Cross-Sectional Studies*, and the *Joanna Briggs Institute Critical Appraisal Checklist for Case Reports* were used for these respective study designs (see Tables 1, 2 for study design type for each paper). The first and second authors rated each study independently, and resolved discrepancies via discussion of individual checklist items, and in consultation with the senior author. The NIH quality assessment tools evaluate the internal validity of studies with consideration of individual checklist items and an overall rating of “good,” “fair,” or “poor”; a higher rating indicates lower risk of bias based on the study design or execution, while a lower rating indicates higher risk of bias. The *Joanna Briggs Institute Critical Appraisal Checklist for Case Reports* provides an overall rating of “include,” “exclude” or “seek further information” based on consideration of eight survey items. Two case reports meeting eligibility criteria received an “exclude” quality appraisal rating, and were excluded from the review. Overall ratings for each study are reported in Tables 1 and 2.

## Results

**Fig. 1:**
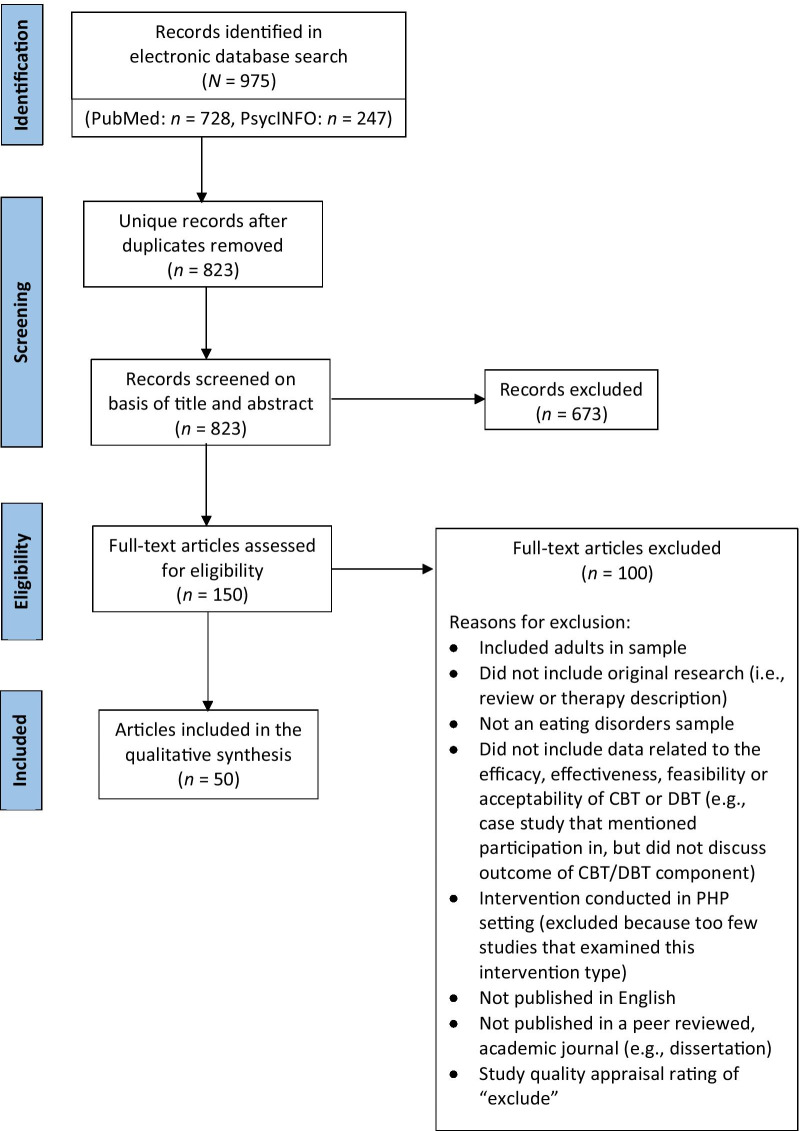
PRISMA flow diagram [25]

The PsycINFO search yielded 209 results for CBT studies and 38 results for DBT studies. The PubMed search yielded 680 results for CBT studies and 48 results for DBT studies. Fifty articles (CBT: *n* = 40, DBT: *n* = 10) met eligibility criteria and were included in this review (see Fig. 1). Tables 1 and 2 provide a complete list of the variables extracted and a summary of relevant findings for each study, including outcome measures.

### CBT for adolescent eating disorders

#### Outpatient settings

Thirty articles examined the feasibility, acceptability, effectiveness, and/or efficacy of outpatient CBT for adolescents, representing 24 different studies (one study produced one secondary analysis article, one study produced two secondary analysis articles, and another produced three secondary analysis articles). Excluding articles that reported data from the same study (*n* = 6), these studies reported outcomes for adolescents with AN (*n* = 11), BN (*n* = 10), BED (*n* = 1), or transdiagnostic eating disorders (*n* = 4) using randomized controlled trial designs (*n* = 7), pre-post designs with no control group (i.e., cohort studies; *n* = 11), an observational cross-sectional design (*n* = 1), and case studies (*n* = 5). About one-half of the original studies (*n* = 13, 54%) reported follow-up data. The majority of studies (*n* = 19, 76%) evaluated individual CBT, a subset of which (*n* = 6, 32%) evaluated CBT-E. Four studies integrated CBT with at least one other treatment type (e.g., FBT, parental counseling, and/or or dietary therapy), and two evaluated group CBT. Most studies (*n* = 18, 72%) reported some form of parental involvement, though this varied greatly from peripheral, “as-needed” involvement (e.g., recruiting parents to help with meal planning; [30]), to formal parent sessions at predefined junctures in treatment (e.g., [31]). The majority of studies examined eating disorder psychopathology (*n* = 22) and/or behaviors (*n* = 15); less than half (*n* = 12) examined weight restoration.

#### AN outcomes

Results of randomized controlled trials and cohort studies (*n* = 10) supported the feasibility and acceptability of outpatient CBT for adolescent AN. Three studies evaluated manualized CBT-E, while other studies evaluated generic forms of CBT adapted for adolescent AN, group CBT, or a CBT module integrated with FBT. Mean treatment attrition was 24% (range: [9–31], *n* = 9), and studies demonstrated higher parent satisfaction with CBT than community-based usual care, [32] but no significant difference in satisfaction between CBT and DBT [33], and no difference in cost between CBT, community-based care, or hospitalization [34]. Effectiveness was partially supported. Statistically significant improvements in weight and/or body mass index was observed (*n* = 4), though the proportion of participants that reached normative weight (i.e., 95% expected body weight) by the end of treatment varied from one third [35] to about one half [31, 36]. While eating disorder psychopathology also significantly improved in the majority of studies that reported this outcome (*n* = 5/7, 71.4%), clinical significance was mixed. Normative levels of eating disorder psychopathology were achieved by the end of treatment for nearly all patients in one study [35], while all patients in another study remained symptomatic at the end of treatment [37]. Multiple studies (*n* = 4) did not report on clinical significance (i.e., effect sizes or interpretation of eating disorder psychopathology scores).

Finally, only two studies used a definition of remission that set a high bar for both cognitive (i.e., global eating disorder psychopathology within community norms) and weight status (i.e., achieved 95% expected body weight, or body mass index equivalent to an adult body mass index ≥ 18.5 kg/m^2^) outcome. These studies suggested that over half of adolescents who completed treatment achieved full remission post-treatment, in CBT-E [31] and an intervention that integrated a CBT module within FBT [36]. Randomized controlled trial data (*n* = 3) found that CBT was neither superior nor inferior to other active treatments (i.e., behavioral family therapy, DBT, inpatient, or community-based care) with respect to eating disorder psychopathology or weight outcomes.

#### BN outcomes

Of the cohort studies and randomized controlled trials that investigated CBT for adolescent BN (*n* = 6), most (*n* = 5) used a manualized version of CBT adapted for adolescents from the Fairburn [38] manual for adult BN, while one study [39] used a manualized version of CBT derived from CBT-E [10], but adapted to the treatment setting [40]. Across studies, acceptability was supported by relatively low attrition (*M* = 14%, range: [0–39]), and effectiveness was supported by clinically meaningful and statistically significant improvement in eating disorder psychopathology. By the end of treatment, two studies reported significant reductions in binge eating and purging [39, 41], while one reported reductions in binge eating only [42]. Both cohort studies found that over half the sample was abstinent from binge eating and purging at the end of treatment [42, 43]. Further, the two studies that reported improvements in eating disorder psychopathology and behaviors (both binge eating and purging) at the end of treatment found that gains were maintained at 3- and 12-month follow-ups [39, 41].

Randomized controlled trial results demonstrated that FBT—compared to CBT—resulted in superior reductions in binge eating and purging at the end of treatment and 6-month follow-up, though outcomes were no longer different between treatment groups at 12-month follow-up [44]. Improvements in cognitive components of eating disorder psychopathology [44], depression, and self-esteem did not differ between CBT and FBT at any time-point [45]. In another study, patients who received CBT showed slightly greater reductions in frequency of binge eating and purging but somewhat less improvement in eating concern at the end of treatment than those who received psychodynamic therapy, while psychodynamic therapy showed a small advantage over CBT in eating concern at the end of treatment [39]. Together, these findings indicate relatively small differences between outcomes with CBT and other active treatments.

#### BED outcomes

One study representing two articles investigated CBT for BED, using a manual for adult BED adapted for adolescents. Attrition rate was 32%, though results showed high levels of therapist treatment adherence and therapeutic alliance [46], supporting feasibility and acceptability. Comparison to a wait-list control group demonstrated that the CBT group had significantly higher rates of abstinence from binge eating, significantly less eating disorder psychopathology, and greater diagnostic remission at the end of treatment [47]. In addition, the CBT group maintained its advantage relative to wait-list control in eating disorder behaviors and psychopathology at all follow-ups through two years.

#### Transdiagnostic outcomes

Two studies combined results for BN and BED; one reported on a transdiagnostic sample of adolescents with AN, BN, or eating disorder not otherwise specified; one reported results for any DSM-5 eating disorder, excluding avoidant restrictive intake disorder. Studies evaluated manualized CBT-E (*n* = 2), CBT adapted (by the research team) for adolescents (*n* = 1), and eclectic approaches that integrated CBT components (*n* = 1). Feasibility and acceptability of the interventions was supported by an acceptable average attrition across three studies (*M* = 28.3%, range: [23–37]) [30, 48, 49] and high post-treatment patient satisfaction in one study [30]. Effectiveness and efficacy were supported by statistically significant improvements in eating disorder psychopathology from pre- to post-treatment with a large effect size in a study evaluating CBT-E [48], and higher rates of abstinence from binge eating at the end of treatment compared to treatment as usual in a study evaluating an adaptation of CBT for adolescents [30]. Further, an effectiveness trial demonstrated that though rate of weight gain was slower in CBT-E compared to FBT at the end of treatment, there were no differences in weight gain between the treatments six months and one year later, and no differences in eating disorder psychopathology or most measures of global psychopathology and clinical impairment at any time point [49]. However, treatment was not assigned but rather chosen by patients and their families, such that baseline differences between treatment groups (e.g., age, duration of illness) were not unexpected. Finally, an observational cross-sectional study found that participants with poor relational/personality functioning did particularly well in eclectic forms of CBT [50].

#### Guided self-help for BN

Five articles representing three different studies (two randomized controlled trials, one cohort study) assessed the feasibility, acceptability, effectiveness and/or efficacy of CBT guided self-help for adolescents, all of which focused exclusively on BN. The interventions were delivered online (*n* = 1), via bibliotherapy (*n* = 1), or compared online and bibliotherapy formats (*n* = 1). Minimal, optional parent involvement was reported in two studies [51, 52]. Variables studied included eating disorder psychopathology, treatment satisfaction, and treatment cost. All three studies included follow-up data. Average attrition across studies was 26% (range: [17–31], *n* = 3). Acceptability was further supported by high adolescent satisfaction with treatment [52], a preference for a self-help format in adolescents wishing to exclude parents from care, and/or seeking increased flexibility and anonymity [53]. Qualitative interview data indicated that guided self-help may be a feasible and acceptable “stepping stone” toward seeking in-person therapy [53]. Feasibility was further supported by lower mean cost of treatment compared to an adapted form of FBT [54].

Statistically significant improvements were reported across all studies in bulimic behaviors (i.e., binge eating, purging and/or fasting; *n* = 3), although rates of remission were modest. One study reported that about half of adolescents had achieved abstinence from behaviors or remission at the end of treatment [55], while another reported that the majority of adolescents remained symptomatic at the end of treatment and follow-up [52]. Two studies made comparisons to another treatment or treatment format. No differences emerged between online versus bibliotherapy guided self-help [55], and compared to family therapy, a significantly higher proportion of participants were abstinent from binge eating at 6-month follow-up in CBT guided self-help, though significant differences between these groups disappeared at 12-month follow-up [51].

#### Inpatient treatment for AN

Five cohort studies assessed acceptability and effectiveness for CBT-based inpatient care, all of which focused exclusively on AN. Three of the studies implemented a CBT component (individual and/or group CBT) within a program with multiple treatment components, while two examined inpatient programs based on CBT-E. The majority (*n* = 5) incorporated formal family sessions. All studies used weight restoration as a measure of outcome, and most (*n* = 5) examined eating disorder psychopathology. Only two studies reported follow-up data. Acceptability was supported by relatively low attrition (*M* = 19%, range: [4–31], *n* = 5). The effectiveness of inpatient programs based on CBT-E was excellent, with nearly all patients (96%) across two studies achieving a normative body mass index at the end of treatment [56, 57]. Results of studies that examined a multimodal treatment program with a CBT component were less strong. Patients across studies achieved statistically significant increases in body mass index, but results were mixed for the proportion who achieved a normative body mass index by discharge. One study found that the majority of patients achieved a normative body weight by the end of treatment [58], while body weight had not reached normative levels, on average, by discharge in two others [59, 60]. Finally, there were statistically significant albeit clinically modest reductions in global eating disorder psychopathology across studies; in two studies, over half of participants continued to have clinically significant eating disorder psychopathology at discharge [57, 60].

### DBT for adolescent eating disorders

#### Outpatient settings

Ten articles representing nine different studies (i.e., one study produced one secondary analysis paper) investigated outpatient DBT for adolescent eating disorders. Across unique studies, DBT was implemented for adolescents with AN (*n* = 1), BN (*n* = 1), BED (*n* = 3), and transdiagnostic eating disorders (*n* = 4). Most studies (*n* = 8, 88.9%) were either cohort studies or case reports, and one was a randomized controlled trial. Interventions implemented full-model DBT (i.e., individual therapy, skills group, and phone coaching; *n* = 2); DBT-informed individual therapy (*n* = 1); DBT skills groups (*n* = 3); and “DBT-informed” FBT (*n* = 3); one study also incorporated an optional parent group [61]. All studies assessed eating disorder psychopathology, two assessed parent reports of eating pathology, and three examined DBT mechanisms of action (e.g., distress tolerance, emotion regulation, DBT skills use). Articles also examined aspects of feasibility and acceptability (*n* = 5), including patient satisfaction, treatment expectations, and therapeutic alliance. Fewer than half of studies (*n* = 2, 20%) reported follow-up data.

#### AN outcomes

Only one outpatient study examined DBT-enhanced FBT in adolescents with AN [62]. Treatment was perceived as “appropriate” and “acceptable” by all patients and their parents with moderate to high ratings of therapeutic alliance, respectively. Acceptability was further supported by a moderate attrition rate (27%). Patients demonstrated significant increases in weight across treatment, with a little over one third achieving weight restoration and over half demonstrating clinically meaningful changes in eating disorder psychopathology at the end of treatment. Small improvements in distress tolerance and emotion regulation also suggested that the intervention was effective in promoting symptom reduction in targeted domains.

#### BN outcomes

One study examined DBT-enhanced FBT in adolescents with BN [63]. Acceptability was highly supported by 100% treatment retention. By the end of treatment, patients reported significant improvements in global eating disorder psychopathology, binge eating, and vomiting, and improvements in parental efficacy and patient emotion regulation were observed.

#### BED outcomes

Four articles representing three different studies examined individual and group DBT for adolescents with BED. Acceptability was supported by 100% treatment retention in all studies. Studies demonstrated high patient satisfaction, with DBT skills groups being rated as feasible, acceptable, and helpful [64, 65]. Patients reported significant decreases in binge eating by the end of treatment in all studies, and 80% of patients no longer met criteria for BED post-treatment in one [65]. Significant decreases in emotional eating [65] and mindless eating [64] were also observed. Notably, Safer et al. [66] found significant reductions in eating restraint, but not weight, shape, or eating concerns, post-treatment. In another study [64], depression scores improved throughout treatment, but anxious and somatic symptoms remained unchanged. Only one study, a case report of DBT-informed individual therapy [66], presented follow-up data and found further reduction in binge episodes by 3-month follow-up.

In studies that examined DBT skills group mechanisms, patients with BED consistently rated radical acceptance as the most helpful skill [64, 67], followed by mindful eating, and three mind states [67]. By the end of treatment, patients showed increases in distress tolerance, cognitive reappraisal, and expressive suppression.

#### Transdiagnostic outcomes

Studies that investigated the feasibility and acceptability of DBT and DBT-informed therapies for transdiagnostic eating disorder samples (i.e., AN, BN, BED, and/or eating disorder not otherwise specified) found that both therapists and patients rated the treatment highly. Across all four studies, acceptability was further supported by relatively low attrition (*M* = 26%, range: [8–33]), and treatment effectiveness was supported by significant improvements in global eating disorder psychopathology. Patients with binge eating and/or purging at baseline either reported significant reductions in [61, 68] or abstinence from these behaviors by the end of treatment [69]. Depressive symptomatology also showed marked improvement post-treatment [69]. Patients demonstrated continued improvement through follow-up, with the majority of patients across studies reporting abstinence from non-suicidal self-injury, binge eating, and purging; and further decreases in global eating disorder psychopathology [61]. One study found large effect sizes for increases in adaptive skills and decreases in dysfunctional coping strategies [68], suggesting high therapist fidelity to the treatment model that effectively targeted the intended mechanisms of change.

Mazzeo et al. [70] performed the only randomized controlled trial, comparing a DBT skills group to a weight management control group for BED and loss-of-control eating. DBT was feasible and satisfactory (although somewhat less so than for weight management), and patients demonstrated similar improvements in global eating disorder psychopathology as those who received weight management. However, the DBT group did not demonstrate improvement in weight concerns, while the weight management group did.

## Discussion

Treatment outcomes for adolescent eating disorders remain modest, highlighting the need for innovative and novel treatment approaches. This review concludes that CBT and DBT-informed interventions have good feasibility and acceptability for adolescent eating disorders, with rates of attrition comparable to FBT trials [8, 71]. Studies also demonstrated high rates of treatment satisfaction, therapeutic alliance, and treatment fidelity, but data establishing effectiveness and efficacy compared to other leading approaches were mixed or incomplete.

The effectiveness of CBT for AN received partial support with mixed results across studies, though efficacy trials demonstrated that CBT for AN was neither inferior or superior to other outpatient (i.e., behavioral family therapy, DBT, community-based care) and inpatient treatments. Support for the effectiveness of CBT was strongest for BN, with consistent, meaningful reductions in binge eating and purging across studies, while efficacy was supported by small differences in outcome compared to other active treatments (i.e., FBT or psychodynamic therapy). CBT guided self-help for BN also led to significant reductions in binge eating and purging and may have enhanced feasibility and acceptability for adolescents who might not otherwise engage in treatment. Meanwhile, weight restoration outcomes were stronger in inpatient programs based on CBT-E for AN relative to eclectic inpatient programs, though replication by alternative research teams would help clarify their feasibility. However, there was a paucity of CBT studies for diagnoses other than AN or BN, and additional effectiveness and efficacy trials are needed across eating disorder diagnoses.

A lack of trials comparing CBT to FBT for AN emerged as a particularly notable gap in the literature, given that FBT is widely considered the first line treatment for adolescent AN but is not always feasible (due to factors such as family capacity/preferences, as well as clinician preferences). Comparisons of FBT to CBT have supported the effectiveness of CBT-E for a majority underweight transdiagnostic eating disorders sample [49], as well as the efficacy of CBT for BN [44]; FBT resulted in faster improvement, but CBT “caught up” with no differences in outcome one-year post-treatment in both studies. Hybrid designs that randomize patients to treatment in real world settings are needed to compare FBT to CBT for AN to clarify their relative efficacy and moderators of treatment outcome. Consistent definitions of remission and attention to the clinical significance of outcomes, particularly in studies of AN, were also lacking and would improve the interpretation of the relative effectiveness and efficacy of CBT versus FBT in future studies.

DBT and DBT-informed treatments demonstrated high levels of feasibility, acceptability, and effectiveness, with reductions in global eating pathology and behaviors across eating disorders. Support for the effectiveness of DBT and DBT-informed interventions was strongest for BN and BED with consistent reductions in or abstinence from binge eating by the end of treatment, and significant reductions in purging for those with BN. The only available randomized controlled trial comparing DBT to weight management found no significant differences in outcomes for BED and loss-of-control eating. Additional randomized controlled trials are thus needed to understand the efficacy of DBT, particularly compared to other treatments over the long-term, given potential differences between treatments that may not emerge in the absence of follow-up data.

Several differences between CBT and DBT emerged. First, contrary to CBT, there were no studies evaluating DBT adapted in inpatient settings. The ability for CBT (particularly CBT-E; [56, 57]) to span inpatient and outpatient settings enables a stepped care approach that may facilitate continuity when transitioning between levels of care (e.g., prevent confusion for adolescents and caregivers due to disparate strategies at different levels of care). While such continuity may be possible in the context of DBT-informed higher level of care programs, these have not yet been studied. Second, though “pure” CBT (i.e., manualized CBT or CBT-E) was evaluated for the treatment of AN and BN, “pure” DBT was evaluated primarily for BED, with DBT skills being integrated within FBT for AN and BN. Indeed, CBT may better target mechanisms hypothesized to maintain AN and BN (e.g., overvaluation of shape and weight), while DBT may be better suited to target mechanisms that maintain psychopathology in BED (e.g., emotion dysregulation). As such, CBT may be more appropriate to compare to FBT than DBT for the treatment of AN and BN, particularly in randomized controlled trials examining mediators of change, as it would not be possible to identify mediators when comparing treatments with overlapping components (e.g., FBT vs DBT integrated with FBT).

Notably, though this systematic review aimed to be comprehensive with the inclusion of non-randomized designs and thoughtful selection of key search terms, it is possible that the keyword search did not encompass all relevant articles, and studies published after December 2020 were not included. The risk of bias assessment revealed multiple limitations across studies: small sample sizes, biased samples limiting generalizability, inconsistent reporting of follow-up data, and inconsistent reporting of elements critical to study quality assessment, including power to detect an effect, and effect sizes. In addition, many studies failed to report race/ethnicity, and the majority of those that did recruited all or majority non-Hispanic White samples. Most studies also recruited majority female or female-only samples. The homogeneity of these samples is an underrepresentation of the diversity in sex, gender identity, race, and ethnicity within the eating disorders population [72–74]. The implementation of treatments also varied considerably across studies, with multiple studies integrating elements of CBT or DBT within other treatments. While this limits conclusions about the specific, unique impact of CBT or DBT, combination treatments may hold promise in enhancing treatment outcome. For instance, FBT combined with cognitive-behavioral techniques may capitalize on the benefit of family supervision and support while also addressing maladaptive cognitions [36]. However, randomized designs are needed to clarify whether CBT may have advantages for certain patients (e.g., through identification of treatment moderators), and whether hybrid forms of treatment (e.g., integrating CBT or DBT with FBT) may enhance patient outcomes.

## Conclusions

CBT and DBT demonstrated good feasibility, acceptability, and preliminary effectiveness across adolescent eating disorders in real-world clinical settings, but their efficacy relative to more established treatments is largely unexamined. Ultimately, the availability of additional evidence-based treatments offers an alternative to FBT, which currently predominates in the treatment of adolescent eating disorders, and is insufficient for some families (e.g., due to family factors such as a history of caregiver abuse, lack of caregiver availability or other barriers to treatment engagement, including caregiver unwillingness possibly affected by poor cultural acceptability and stigma related to mental health and mental health treatment; or co-occurring disorders and/or other psychopathology better managed within a DBT framework).

New treatments or even existing treatments blended with new treatments may enhance outcomes for difficult-to-treat populations or reduce risk of relapse. We recommend that future research: (1) use adequately powered, randomized designs with representative transdiagnostic eating disorder samples under real-world conditions to compare CBT and DBT to other leading treatments (including FBT); (2) evaluate the relative utility of “pure” versus integrated/hybrid forms of treatment (e.g., the efficacy of broad CBT-E vs CBT integrated with FBT); (3) assess treatment moderators to better guide treatment matching; (4) evaluate mediators of treatment response, and reasons for non-response to inform treatment modifications that improve efficacy and efficiency; and (5) improve the consistency of reporting elements critical to study quality assessment—power and effect sizes—to improve the interpretation of outcomes. Advancing research on alternative treatment options is imperative given the serious consequences of eating disorders [4], relative likelihood for a severe and enduring presentation [75, 76], and remission rates for leading treatments.

## Data Availability

The datasets used during the current study are available from the corresponding author on reasonable request.

## Abbreviations

AN: Anorexia nervosa
BED: Binge eating disorder
BN: Bulimia nervosa
CBT: Cognitive behavior therapy
CBT-E: Enhanced cognitive behavior therapy
DBT: Dialectical behavior therapy
FBT: Family-based treatment
NIH: National Institute of Health
